# Suit Up: A Systematic Review of the Personal Protective Equipment (PPE) Recommended and Utilized by Various Classes of Responders to Nuclear Radiological Disasters at Nuclear Power Plants

**DOI:** 10.1017/S1049023X23006672

**Published:** 2024-02

**Authors:** Chaverle K. Noel, Erica D. Bruce, Benjamin J. Ryan

**Affiliations:** 1. Department of Environmental Science, Baylor University, Waco, Texas USA; 2. Frist College of Medicine, Belmont University, Nashville, Tennessee USA

**Keywords:** emergency, nuclear power, personal protective equipment, preparedness, radiation

## Abstract

**Introduction:**

Interest in nuclear power as a cleaner and alternative energy source is increasing in many countries. Despite the relative safety of nuclear power, large-scale disasters such as the Fukushima Daiichi (Japan) and Chernobyl (Ukraine) meltdowns are a reminder that emergency preparedness and safety should be a priority. In an emergency situation, there is a need to balance the tension between a rapid response, preventing harm, protecting communities, and safeguarding workers and responders. The first line of defense for workers and responders is personal protective equipment (PPE), but the needs vary by situation and location. Better understanding this is vital to inform PPE needs for workers and responders during nuclear and radiological power plant accidents and emergencies.

**Study Objective:**

The aim of this study was to identify and describe the PPE used by different categories of workers and responders during nuclear and radiological power plant accidents and emergencies.

**Methods:**

A systematic literature review format following the PRISMA 2020 guidelines was utilized. Databases SCOPUS, PubMed, EMBASE, INSPEC, and Web of Science were used to retrieve articles that examined the PPE recommended or utilized by responders to nuclear radiological disasters at nuclear power plants (NPPs).

**Results:**

The search terms yielded 6,682 publications. After removal of duplicates, 5,587 sources continued through the systematic review process. This yielded 23 total articles for review, and five articles were added manually for a total of 28 articles reviewed in this study. Plant workers, decontamination or decommissioning workers, paramedics, Emergency Medical Services (EMS), emergency medical technicians, military, and support staff were the categories of responders identified for this type of disaster. Literature revealed that protective suits were the most common item of PPE required or recommended, followed by respirators and gloves (among others). However, adherence issues, human errors, and physiological factors frequently emerged as hinderances to the efficacy of these equipment in preventing contamination or efficiency of these responders.

**Conclusion:**

If worn correctly and consistently, PPE will reduce exposure to ionizing radiation during a nuclear and radiological accident or disaster. For the best results, standardization of equipment recommendations, clear guidelines, and adequate training in its use is paramount. As fields related to nuclear power and nuclear medicine expand, responder safety should be at the forefront of emergency preparedness and response planning.

## Introduction

The Three Mile Island disaster of 1979 (Pennsylvania USA), Chernobyl meltdown of 1986 (Ukraine), and Fukushima Daiichi disaster of 2011 (Japan) elicited some skepticism in the nuclear power industry. These events highlighted the need for worker safety at nuclear power plants (NPPs). In response, agencies such as the Nuclear Regulatory Commission (NRC; Rockville, Maryland USA) and the International Atomic Energy Agency (IAEA; Vienna, Austria) have implemented strict regulations. These include engineering and administrative safety mechanisms at powerplants, and occupational personal protective equipment (PPE). These PPE include clothing or specialized equipment and tools designed to protect workers from exposure to ionizing radiation through shielding and preventing contact with contaminated particles and liquids.^1,2^ As nuclear power advances, modular nuclear reactors increase, and nuclear medicine expands, the need to better understand PPE requirements is rapidly increasing.

The United Nations Scientific Committee on the Effects of Atomic Radiation (UNSCEAR; Vienna, Austria) reported on both the Chernobyl and Fukushima Daiichi disasters. Twenty-eight first responders at Chernobyl perished due to radiation exposure. Another 240,000 clean-up workers and liquidators were deployed to the “hot zone” of 30km surrounding the reactor, exposing them to 100mSv of radiation. An additional 600,0000 civilians and military members were drafted for heavy remedial activities until ∼1990.^3,4^ The Fukushima event resulted in an atmospheric release of 100-500PBq of Iodine-131 and 6-20PBq of Caesium-137 radionuclides. During this response, the 100mSv occupational exposure allowance was increased to 250mSv for responders. An estimated 25,000 workers from the Japanese Self-Defense Force, Coast Guard, and firefighters carried out initial mitigation efforts. Tens of thousands more municipal workers also responded, and members of the United States Military assisted in supporting roles and radiation monitoring. It is estimated that the average effective dose for the 25,000 workers was 12mSv, but some exceeded 100mSv.^5,6^

While information about the Chernobyl event is not readily available, literature outlines the PPE utilized for Fukushima responders. Technical workers wore double-layer Tyvek protective coveralls and tight fitting, full-face respirators with P100 filters. High boots were required with vinyl shoe coverings and double gloves (cotton and rubber). Whole-body counters were eventually added to the ensemble. The low inventory of alarming pocket dosimeters owing to the preceding tsunami resulted in only the team lead wearing one. Workers’ exposures were estimated based on the readings from the team lead’s unit.^7^

This paper aims to build on these lessons and experiences by identifying and describing the PPE used by different categories of workers and responders during nuclear, radiological power plant accidents and emergencies. It was conducted without external funding, and with kind support of Baylor University (Waco, Texas USA). The findings will help understand how the PPE used by these workers and responders may differ while protecting their health and well-being. This information is vital to balance the tension between a rapid response, preventing harm, protecting communities, and safeguarding workers and responders. Ultimately, this will help inform PPE needs for future responses and emergency preparedness planning for nuclear, radiological power plant accidents and emergencies.

## Methods

A systematic literature review of five databases (SCOPUS [Elsevier; Amsterdam, Netherlands], PubMed [National Center for Biotechnology Information, National Institutes of Health; Bethesda, Maryland USA], EMBASE [Elsevier; Amsterdam, Netherlands], INSPEC [Institution of Engineering and Technology; United Kingdom], and Web of Science [Clarivate Analytics; London, United Kingdom]) was conducted in June 2022. Selection was limited to papers published in English or with English translations. The search terms utilized were: [(PPE OR ‘personal AND protective AND equipment’ OR ‘personal AND protection AND equipment’ OR ‘RPE’ OR ‘Radiological AND Protective AND Equipment’) AND (Nuclear)]. Articles were included if they identified PPE for radiological or nuclear activities. If an article did not identify articles of PPE or was not about nuclear radiological events, it was excluded. The Preferred Reporting Items for Systematic Reviews and Meta-Analyses (PRISMA) 2020 guidelines were used to conduct this review. This methodology was selected for its scientific strength, thoroughness, and ability to produce a clear, concise, and unbiased outcome.^8^

Once the articles were retrieved, Covidence (Covidence; Melbourne, VIC, Australia) systematic review management tool was used to screen title and abstracts, followed by a full-text review. Data were extracted for this review from the articles that made it through those initial stages. The results were sorted according to the types of PPE used in the articles, the class of responder activated, and which phase of the emergency they responded in. Any examples of PPE efficacy identified were also extracted to establish trends in the type of PPE utilized and the level of efficacy offered. Microsoft Excel, Version 2306 (Microsoft Corp.; Redmond, Washington USA) was used for data visualization. An interdisciplinary team of individuals from public health, environmental science, and toxicology backgrounds reviewed the articles to ensure thoroughness and eliminate bias.

## Results

**Figure 1.**
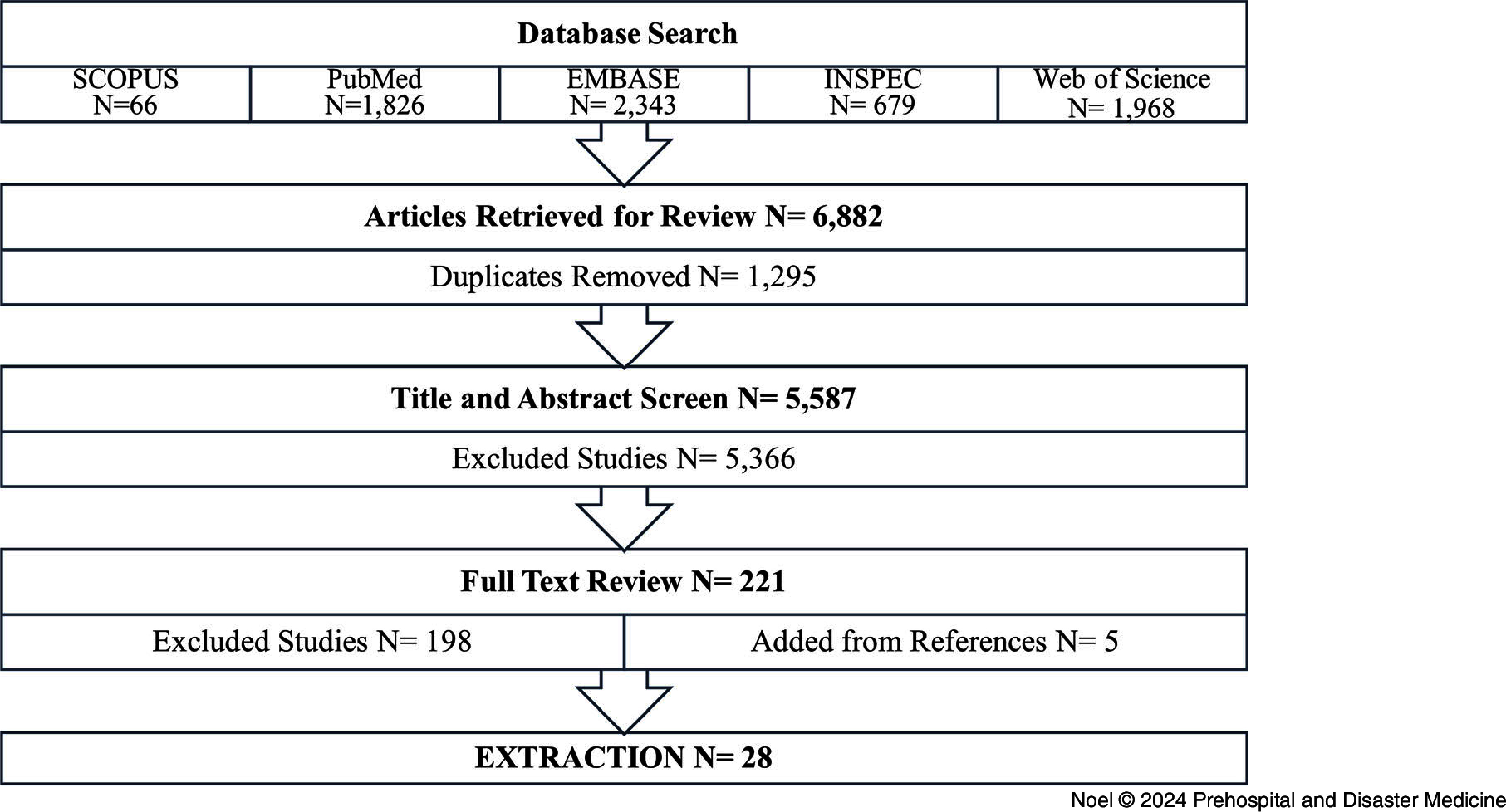
Flowchart of the Literature Review and Article Selection Process.

The search terms yielded 6,882 publications. After removing the duplicates, 5,587 potentially relevant publications moved on to the screening phase. The titles and abstracts were screened, followed by the full texts, to assess whether they met the inclusion criteria. Twenty-three articles were eventually selected from this search, and five publications were added manually due to their relevance to the topic. The selection process produced 28 publications for review and extraction (Figure 1). A summary of the articles reviewed is show in Table 1.^1,2,9–34^

**Table 1.** Summary of the Articles Reviewed

Ref #	Author	Setting/Summary	Responder Classification	Response Phase	PPE Recommended/Used
14	Chen and Demachi	Fukushima Daiichi Atomic Power Plant decommissioning; utilization of a vision-based automated system for monitoring PPE adherence	Decontamination/ Decommission Team	Accident/ Decommissioning	Head Covering, Mask, Gloves, Protective Suit, Coverall, Foot Covering
33	Mortelmans	Survey of Dutch hospitals preparedness for treating patients in the event of a CBRN accident	EMS	Accident/ Preparedness Training	Respirator, Protective Suit
34	Eichorst and Clay	Evaluation of contamination events at two similar Department of Energy (DOE) research sites	Plant Workers	Everyday Work	Head Covering, Eye Covering, Respirator, Gloves, Protective Clothing, Coveralls, Shoe Covering
26	Millard and Vaughan	Two studies of gloves used with air-fed suits at decommissioning sites, or containment four microbiological work- only nuclear workers assessed for this paper	Decontamination/ Decommission Team	Everyday Work/ Decommissioning	Gloves, Protective Suit
25	Kozlovaska, et al	Reviewed the shielding and protective properties of common radiological protective clothing	Plant Workers/ EMS	Accident	Head Covering, Face Covering, Protective Suit, Vest, Shoe Covering
18	Canu, et al	Examination of PPE compliance of workers the hazardous nuclear fuel industry	Plant Workers	Everyday Work	Ear Covering, Eye Covering, Mask, Overall
13	McCall	Report on the Chernobyl disaster of 1986 from a former plant worker	Security Guard	Accident/Active Response	Respirator, Protective Clothing
2	Vosahlikova	Evaluation of ease of decontamination of protective suits against aerosolized radiological contamination	Decontamination/ Decommission Team	Accident/ Preparedness Training	Protective Suit
27	Millard, et al	Literature review; observations at decommissioning sites; physiological evaluation of air-fed suits in different environments	Decontamination/ Decommission Team	Everyday Work/ Decommissioning	Head Covering, Gloves, Underwear, Protective Suit, Foot Covering
30	Khaloo	Review of dosimetry data derived from projects at off-shore CANDU nuclear power plants completed by maintenance workers from a Canadian agency	Plant Workers/ Support Personnel	Everyday Work/ Decommissioning	Dosimeter, Respirator
23	Grugle and Kleiner	Reviewed the effects of protective gear on military and civilian teams in CBRN response	Military	CBRN	Head Covering, Mask, Gloves, Protective Suit, Shoe Covering
10	Yasui	Fukushima Daiichi Atomic Power Plant post-radiological event; investigation of claims of dosimeter tampering and efforts to suppress exposure readings	Decontamination/ Decommission Team	Accident/ Decontamination	Protective Suit, Dosimeter
12	Yasui	Fukushima Daiichi Atomic Power Plant post-radiological event; worker exposure from inappropriate use of PPE-issues with controlling and managing worker exposures	Decontamination/ Decommission Team	Accident/ Decontamination	Respirator, Protective Suit, Dosimeter
9	Yasui	Fukushima Daiichi Atomic Power Plant post-radiological event; worker exposure from inappropriate use of PPE-steps taken to address these issues	Decontamination/ Decommission Team	Accident/ Decontamination	Coverall, Vest, Foot Covering
11	Yasui	Fukushima Daiichi Atomic Power Plant post-radiological event; presentation of new regulations implemented by Japanese government in response to the event	Decontamination/ Decommission Team	Accident/ Decontamination	Masks, Protective Suit
24	Schumacher	Difficulties faced by paramedics attempting bag-valve-mask ventilation during response to CBRN events	Paramedic	Active Response to CBRN Event	Respirator
17	Cumo, et al	Examination of the physiological factors that affect nuclear and other harsh environment workers while wearing PPE	Plant Workers	Decommissioning	Protective Suit
32	Musolino, et al	Theoretical/lab-based modelling of efficacy of Preventative Radiological/Nuclear Detectors (PRND) post-unplanned radioactive material release	First Responders	CBRN/Nuclear Accident	Dosimeter
31	Yoshitomi and Kowatari	Modelling of the radiation dose to the eyes at nuclear facilities, and whether different PPE use influences this dosage	Plant Workers	Everyday Work	Eye Covering, Respirator, Apron
16	McWhan and Dobrzynska	Monitoring of eye lens dosage of radiation in the nuclear industry in response to new limits set by the European Union	Plant Workers	Everyday Work	Dosimeter
15	Ono	Status update of decontamination and decommissioning efforts at the Fukushima Daiichi site 10 years post-event	Decontamination/ Decommission Team	Accident/ Decontamination	Respirator, Dosimeter
1	IAEA	Technical manual providing guidelines for radiological industry professionals on radiation protection	Plant Workers	CBRN	Respirator, Gloves, Protective Suit, Apron, Foot Covering
19	Kozlovaska, et al	Modelling and measuring of protective qualities of PPE designed for x-ray and gamma-ray radiation exposure	Not Specified	Accident/ Preparedness Training	Protective Suit, Vest
28	Gikiewicz and Bralewska	Literature review of factors influencing PPE selection for rescue personnel during CBRN events	EMS, Plant Workers	CBRN	Eye Protection, Ear Protection, Respirator, Gloves, Protective Suit, Vest, Shoe Covering
20	Hiraoka	Literature review; presentation of health issues in workers stemming from exposure at Fukushima Daiichi accident	Support Personnel, Military, EMS, First Responders	Accident/ Decontamination	Respirator, Gloves, Coverall, Vest, Dosimeter
29	Rissanen and Rintamäki	Examination of nuclear, biological, and chemical PPE protective capacity, under cold conditions	Not Specified	CBRN	Head Covering, Mask, Gloves, Underwear, Protective Suit, Coverall, Foot Covering
22	Bandera, et al	Just-in-time training proposal for support personnel mobilized after an emergency CBRN event	Support Personnel	Accident	Eye Protection, Respirator, Gloves, Protective Suit, Coverall, Foot Cover
21	Likhtarev	Workers involved in decommissioning work (strengthening the Sarcophagus) at Chernobyl	Decontamination/ Decommission Team	Decommissioning	Respirator, Mask, Protective Suit, Coverall

Abbreviations: PPE, personal protective equipment; CBRN, chemical, biological, radiation, nuclear; EMS, Emergency Medical Services.

As one of the most recent events, the Fukushima Daiichi NPP disaster was most prominent in the articles (n = 7).^9–15^ Most of the other articles reviewed provided theoretical PPE recommendations for accidents based on preparedness training events, identified everyday PPE for NPPs, or were classified as decontamination and decommissioning events (n = 13).

### Categories of Workers Involved in NPP Response Work

Many different types of workers were required when responding to the nuclear and radiological events. These included workers who are on site every day and those involved in decontamination, skilled support personnel, military, and medical professionals. The on-site workers were found to be the first response due to their proximity and were more likely to be exposed to ionizing radiation if proper PPE was not worn.^1,16–20^ Decontamination workers and teams often supported the on-site workers post-event. Chernobyl decontamination and decommissioning workers cleared the work area, prepared the workspace, and conducted electrical welding, assembly work, metal cutting, and boring, battering, and drilling.^21^ Similarities in Fukushima Daiichi decontamination workers also emerged, as they installed electrical cables, drained contaminated water from tanks, changed out water filters, removed rubble, retrieved debris, and concretized and paved surfaces.^9,14,15^

Skilled support workers were also essential during this response phase. Although these workers did not typically work in the NPP setting, or in nuclear radiological spaces, their skillsets were critical after an emergency.^22^ They included laborers, ironworkers, carpenters, operations engineers, utility workers, sanitation workers, and administrative staff.^20,22^ Members of the military and self-defense forces were also on site in various capacities. These generally included rescue missions, resident evacuations, cooling the nuclear reactor (alongside other first responders), and monitoring of exposure levels.^4,20^ They were often included in emergency medical technician/EMT teams as well.^23^ Other medical professionals responding included paramedics and the Emergency Medical Services (EMS).^20,24^

### PPE Use and Recommendations

**Figure 2.**
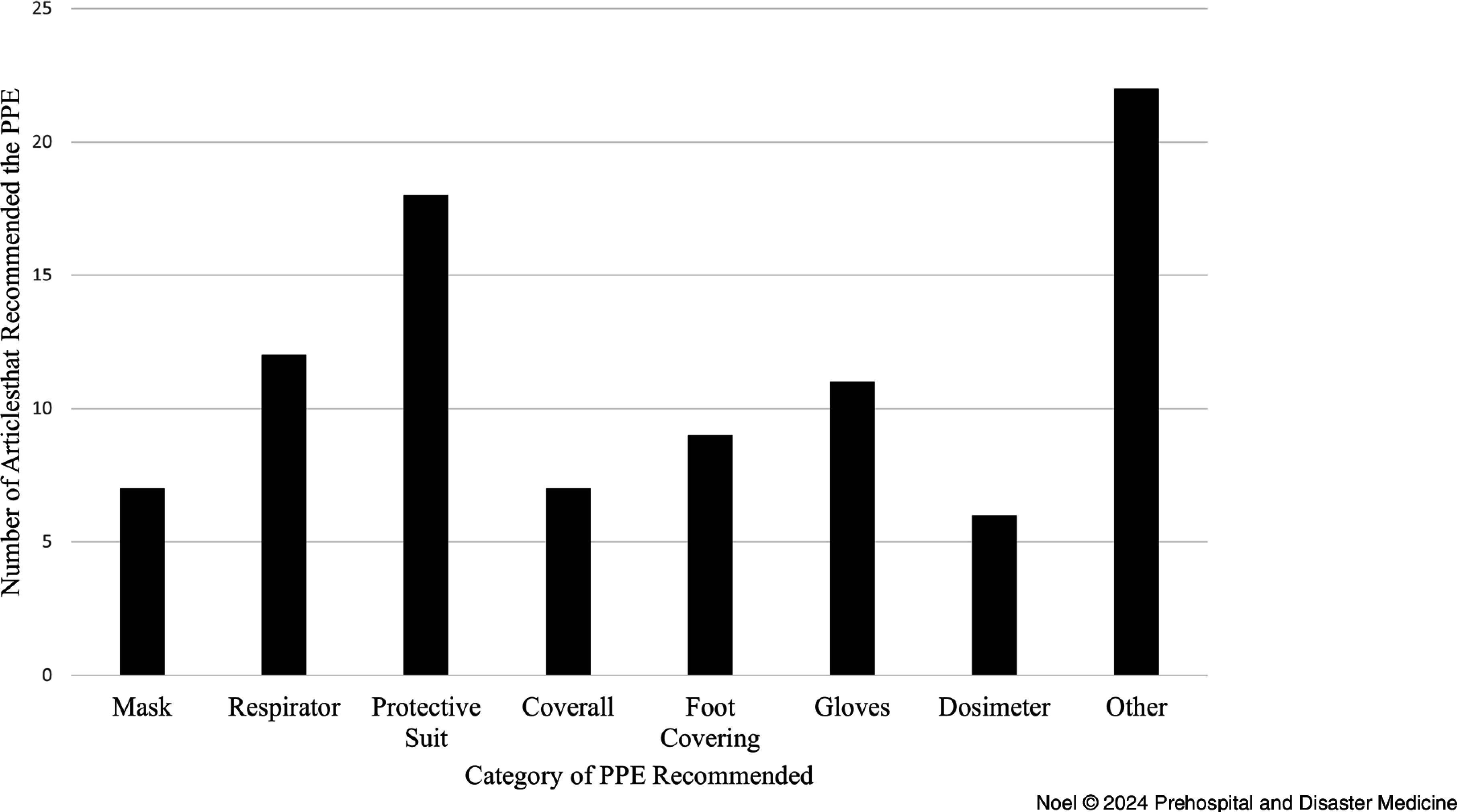
Categorization of the PPE Recommended in the Articles Reviewed. Abbreviation: PPE, personal protective equipment.

Protective suits or whole-body coverings, respiratory equipment, and gloves emerged as the most used or recommended pieces of PPE. Protective suits presented in 19 of the 28 articles reviewed. Gloves were next, followed by varying types of respirators, then foot covering. While the “other” category peaked with 22 recommendations (Figure 2), this category included PPE which were not recommended frequently (average ≤ three times). Each of these are discussed below.

### Protective Suits and Coveralls

The IAEA established four classifications of protective suits, distinguished by their performance levels (A-, B-, C-, and D-Suit; Table 2). The A-Suit was non-ventilated, not pressurized, and was made from a permeable or non-woven fabric. This suit offered the lowest level of protection. The C-Suit and D-Suit provided much higher levels of protection, as they were ventilated, impermeable, and included respiratory protective equipment (RPE).^1^

**Table 2.** Comparison of the Classes of PPE Suits According to the IAEA

Suit Classification			Category A Suit	Category B Suit	Category C Suit	Category D Suit
**Prospective Surface Contaminant**	Solid	Low	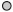	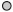	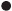	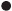
		High		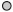	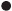	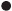
	Liquid	Low		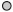	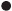	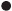
		High	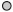	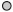	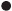	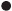
**Prospective Airborne Contaminant**	Aerosol	Weak		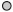	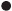	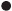
		High			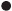	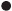
	Gas	Weak		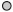		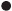
		High				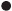

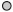
 These suits must be used with appropriate, additional respiratory gear.

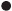
 These suits do not require additional respiratory gear.

Note: Table is a recreation of a published IAEA illustration.

Abbreviations: PPE, personal protective equipment; IAEA, International Atomic Energy Agency.

Protective suits appeared to be a key part of the PPE required for NPP and chemical, biological, radiological, and nuclear (CBRN) events. However, the literature revealed large variability in the types of protective suits available for responders and the level of protection they provided. A combination of one- and two-piece protective suits, with and without hoods, were most common. Disposable versus reusable suits made from different materials, and of varying densities, were also evident. Coveralls and overalls for full-body protection were common, and in many cases, coveralls were donned over the protective suits.

The accident and decontamination articles saw workers wearing full-body suits. This included halos to supply air or full-face respirators. One article reviewed suits with bio-rubber densities ranging from 0.053g cm^−2^ to 2.08g cm^−2^.^25^ For extra shielding properties, lead and tungsten were incorporated into some suits.^25^ Millard and Vaughan found either a one-piece or two-piece suit was deemed appropriate for the job.^26^ However, the one-piece suits required two additional over-suits and an over-hood to complete their PPE ensemble, but one additional over-suit and no over-hood was required for the two-piece protective suits.^26^ Yasui also saw workers wearing a Type-C hazmat suit for their decontamination work.^11^ This hazmat suit was only required if the ambient dust level at the site was greater than 10mg/m^3^ and the radioactivity level was more than 500,000Bq/kg. Where the contamination did not meet those requirements, no suit was required.^11^ A two-piece ensemble was also worn by some EMS responders. Military EMS responders donned a protective jacket and pants over-garment atop their base EMS attire.^23^

### Respirators and Masks

The IAEA manual noted that RPE may be separated into two categories: respirators and breathing equipment. The respirators removed or filtered particulate matter and included half- and full-face masks, filtering face respirators, and those respirators which were equipped with a fan and filters that circulate air through protective suits, headgear, or masks.^1^ The use of masks appeared frequently in decommissioning and decontamination activities. These recommendations varied from half- to full-face respirators and canister respirators.^21,26,27,35^ Masks (including those made of non-woven textiles and disposable dust masks) were recommended in settings where the contamination was dust particles, particulate matter, or only probabilistic in nature.^11,14,27^ Fukushima Daiichi decontamination workers wore non-woven or disposable masks in low-contamination settings.^11,15^ Where the ambient dust concentration was 10mg/m^3^ or lower, and the contamination in the soil being tilled was 50,000Bq/kg or below, this type of mask was deemed sufficient. A step-up to an 80% filtration efficiency mask replaced the non-woven ones when site conditions exceeded 10mg/m^3^ or the 50,000Bq/kg threshold. This was further increased to 95% filtration efficiency mask or greater if both the ambient dust and the soil contamination exceeded the lower threshold.^11^ Canu, et al also differentiated the types of masks required as the workers’ hazard increased. The recommendation went from complete masks or P3 half-masks to complete masks with specific filtration cartridges as the workers exposures varied from fibers to metal dusts, to chemical byproducts and Uraniferous products.^18^ While there was wide-spread RPE use or recommendations throughout the literature, issues with worker adherence in this category frequently appeared. Fukushima case reports found 17 workers were exposed to between 100-250mSv of internal radiation doses owing to respirators that were not fitted properly or guidelines that were not adequately followed.^12^ Canu, et al also reported adherence gaps among Uranium workers, as they reported that sometimes they did not feel the need to wear their masks.^17^

### Gloves

Glove selection needed to be appropriate for the job and level of exposure involved.^28^ For example, two pairs were required for workers with lower probability of exposure and four pairs for workers with higher probability of exposure.^27^ The PPE ensemble for plant workers (and relevant responders) with higher exposures included a cotton liner as a base, followed by several layers of chemical or barrier gloves, and a cut resistant or a specialist glove on the outside.^11,27,29^ Decommissioning activities often included handling sharp objects, so extra cut-resistant gloves were recommended as the top layer of their multi-glove ensemble.^26^ In CBRN events, chemical resistant gloves were evident.^28^ Multiple layers also provide physiological properties. One article mentioned that the cotton base layer acted as thermal insulation under rubber gloves for some responders.^29^ Despite many articles noting the need for gloves, one article indicated that gloves worn by military responders were adjusted frequently.^23^ A decrease in manual dexterity was also reported due to glove use. This was especially evident where tasks required fine motor skills.^27^

### Foot Covering

While many of the full-body protective suits covered the feet of those wearing it, foot covering recommendations included additional specialized foot covering equipment. Military responders in one article were required to don additional over-boots over their gear to respond to these radiological events.^23^ Millard and Vaughan specified that their decontamination teams were required to wear Wellington boots with their protective suits.^26^ One of Yasui’s articles hinted at the implications of workers not wearing appropriate footwear. They noted two cases of contamination where workers wore short rubber boots where long boots would have been appropriate due to their work with contaminated water.^9^

### Dosimeter

Dosimeters allowed workers to be aware of their personal exposure or the ambient levels of exposure on site. Generally, whole-body dosimeters were acceptable (especially in the nuclear industry, and for non-medical industries).^16,30^ While some activities (like decommissioning work) may subject responders to higher eye doses, the estimate provided from whole-body dosimeters remained accurate.^16,30^ Alarming dosimeters were the preferred device for monitoring exposure.^12,20,30^ During the Fukushima response, many dosimeters were damaged from the tsunami and led to grouping of the staff and issuing whole-body counters to the team lead to serve as the exposure dose for the group.^11^ Eventually, enough whole-body counters were attained for the group.^11^

### “Other” Items of PPE

Other items included articles of clothing like underwear and vests, and gear like head, ear, and eye coverings. Two articles included underwear as the base layer before donning varying types of full-body protective suits in their work.^27,29^

Different types of vests were also mentioned in four articles. One case report from Fukushima noted that tungsten vests were part of the PPE used in areas where radiation doses rate was high.^11^ Cooling vests were also donned by some Fukushima workers in extreme heat conditions.^20^ The other two articles performed simulation exercises to explore the protective qualities of four different vests (one vest with tungsten dispersed in resin, two bio-rubber vests of different thicknesses, and one Demron [Radiation Shield Technologies; Coral Gables, Florida USA]).^19,25^ They established that these vests were generally suitable in case of nuclear facility accidents or other radiological emergencies, but as beta or gamma particle exposure increased, the level of attenuation of the radionuclides decreased.^19,25^

A lead apron could also reduce workers’ cumulative exposure in the medical and nuclear sectors. However, its protective qualities also decreased as the exposure to beta or gamma particles increased.^31^ The protective ability of aprons was also identified where workers perform tasks with heat or power tools that may generate debris. Donning an apron over an air-fed suit reduced the probability of damaging the suit. However, this extra layer would increase the physiological burden on the wearer or increase entry time.^27^

Head coverings were apparent in six articles. Fukushima responders used hard hats in addition to hooded coveralls and respirators in high-contamination zones.^14^ The head coverings in the other five articles were combinations of hoods and masks, or hoods as part of the protective suit or coveralls that were worn by responders.^23,25–27,29^ One article assessed Mission Oriented Protective Posture (MOPP) military ensembles. The MOPP-4 ensemble included a mask/hood combo that was donned by military personnel for emergency response that covered the head.^23^ Millard and colleagues reviewed air-fed suits that were worn with additional over-suits. This provided multiple hoods from the air-fed suits and the oversuits.^26,27^ Whole-body radiation shielding clothing protective suits with integrated hoods were touted as appropriate for special shielding in several emergency settings. However, once the energy exposure was greater than x-rays, the attenuation properties decreased significantly (less than 30%).^25^ Rissanen and Rintamäki also mentioned the use of full-body suits with hoods.^29^ This article specified a one-piece impermeable suit with butyl rubber hood and a semi-permeable suit which included a jacket with an attached hood.^29^

Earmuffs and earplugs were specified for nuclear fuel workers, and were recommended for workers of the European Gaseous Diffusion Uranium Enrichment Consortium (EURODIF; Pierrelatte in Drôme, France).^18^ However, the article noted that workers cited that they did not always feel like they needed these PPE, and that the earmuffs and earplugs were a hindrance or were uncomfortable. This led to six percent of the workers surveyed having stated that they never wear either of these ear protections, and eleven percent and nine percent, respectively, saying that they only wear them occasionally.^18^

Lastly, the utilization of eye protection in the form of splash-proof goggles, safety glasses, and face shields were noted as part of the training on protective gear for support personnel to CBRN events.^22^ Protective glasses were helpful in shielding the eye lenses from high-exposure doses.^31^ Canu also echoed the requirement of protective goggles for fuel workers. However, these anti-spray googles were reported among the least used PPE by the workers. The inability to find them and the perception that they were not necessary were cited as the reason for the lack of use. Workers who wore prescription lenses also reported issues adhering to this requirement.^18^

### Tools and Equipment

The IAEA radiation manual introduced special equipment in addition to clothing that may act as PPE.^1^ These should increase work efficiency and reduce exposure time. One article saw decontamination workers provided with work aids and a range of tools that were appropriate for their tasks. These included ergonomic aids like platforms where the height could be adjusted. Drums or waste containers for debris to decrease carrying time and distance were used, as well as brushes, brooms, and scaffolding poles. “Small tools or objects” were also broadly suggested as aids.^27^

## Discussion

Response to a NPP accident or other large-scale radiological facility requires many types of responders and timelines. This may span from within the first few seconds of the event to years later. The immediate response, which occurs within seconds of the event, is by plant workers. After this, the early stage of crisis response often falls under local jurisdiction.^32^ This could include local responders such as law enforcement, fire, and EMS. Initial incident response should include radiation detection, dosimetric data collection, and implementation of community-wide protective mechanisms. Within 24 to 36 hours post-event, federal-level response would generally become activated if local responders are incapable of responding effectively.^32^

The Fukushima disaster saw workers recruited from other power plants, adjacent industries, and community volunteers. This brought communication barriers, shortages of adequate PPE, issues monitoring exposure levels, and external workers and contractors to be integrated into a system already thrown into a tailspin by the disaster.^10–12^ Setting baseline, standardized PPE requirement for NPP accidents would establish a starting point across the board for worker safety. This can easily be modified up or down with additional layers, if necessary, but at the base level, this would ensure sufficient protective factors for responders.

Workers are required to wear multiple layers of PPE, especially during decontamination. This often includes air-fed or whole-body suits encapsulating the hands and feet, with additional boots, shoe coverings, several layers of gloves, and overalls donned over this full-body covering. These layers provide the best protection from suit penetration and subsequent contamination in emergency settings.^11,26,27,29^ The consequences of insufficient gear or ill-fitting gear is significant. For example, several cases of contamination occurred at Fukushima due to workers wearing half-boots instead of long protective boots when carrying out work in contaminated water.^9^ Worker contamination from water falling on an employee’s head and another becoming soaked from a hose due to their lack of liquid-proof coveralls was also evident.^9^ Excess exposure to radionuclides owing to masks not properly fitted for the responders also occurred.^9^ For these reasons, proper training on PPE use, especially in emergency scenarios, is essential for new employees, along with refreshers for existing employess.^26,27^ Timely outfitting of employees with items such as masks or respirators which require molding is also important. This ensures that employees know what gear is appropriate for use in emergency settings, know how to accurately don and doff it, and can be prepared for its physiological burdens.^26,27^

Many of the examples of contamination mentioned in the articles stemmed from lack of adherence to the PPE requirements on site or the need for additional PPE. Yoshitomi and Kowatari noted that while full-face respirators may provide some shielding properties to the eyes, it may not be suffiecient.^31^ Kozlovska, et al also noted that the protective qualities of some of the gear available for responders decreased depending on the level and length of their exposure.^19^ To compound this issue of insufficiency, workers do not always don nor doff their gear successfully or completely.^9–12,27^ While training is appropriate to address some of these issues, it establishes a need to also enforce the safety requirements. There is a need for better monitoring of worker adherence to the guidance. One possible solution could be automated and vision-based software to carry out this task.^14^ The presence of a safety officer on site to enforce safety codes (especially during emergencies) and central placement of site guidelines for emergency response is also imperative.

The physiological burden of PPE and its effect on worker efficacy must not be overlooked. While in many cases the articles justified the need for the PPE, many of them enacted a physiological burden. This manifested as loss of dexterity, overheating or increased sweating, and an increase in task completion time.^20,23,26,27,29^ To decrease these effects, several authors noted that workers should be fit in all aspects for this type of work. This included ensuring mental, medical, and physical states of wellness.^17,27^ Proper training on the use of PPE and tabletop exercises and drills will help ensure responder preparedness in case of an emergency. This would also strengthen emergency preparedness by increasing confidence in the processes used to protect against harmful radiation during response and recovery efforts.

## Limitations

Many variations of acronyms for PPE are used in the NPP and CBRN industry, such as RPE and RSPC (Radiation Shielding Protective Clothing). The authors limited the search terms to PPE and RPE. These were the most common terms used by the governing bodies such as the IAEA and NRC to denote clothing, special equipment, and tools that provide protective qualities. Conversely, several alternative uses of the acronyms PPE and RPE such as Retinal Pigment Epithelium emerged from unrelated fields. The exclusion criteria were applied strictly to eliminate such articles. Documents that were available in English or with English translations were a requirement for this review. This review also encompassed case reports published by the governments. While some of these findings were apparent in other scientific articles, careful consideration of its credibility and aptness may be warranted.

## Conclusions

As the prevalence of nuclear power advances, the growth of modular nuclear reactors increases, and the utilization of nuclear medical services continues to expand, the need for clear PPE guidance is paramount. Incorporating lessons learned from events such as Chernobyl and Fukushima into emergency preparedness and response planning can mitigate the effects of nuclear and radiological power plant disasters.^1^ This review highlighted that numerous classes of workers respond in different phases to various nuclear radiological events.^1,9,14–22,24,32^ Selection of gear that is appropriate for the type of accident, paired with efficient donning and doffing practices, minimizes responders’ exposure to ionizing radiation.^1,25–27^ Clear guidelines, reinforced with adequate training and frequent drills for PPE use, will help to achieve this goal.^9,25–27^ There is also a need to harmonize PPE requirements and standard operating procedures for responders and workers based on substantive data. This would strengthen emergency preparedness by increasing confidence in the processes in-place to prevent harmful radiation exposure during response and recovery efforts.
